# Evaluating the performance of large language models: ChatGPT and Google Bard in generating differential diagnoses in clinicopathological conferences of neurodegenerative disorders

**DOI:** 10.1111/bpa.13207

**Published:** 2023-08-08

**Authors:** Shunsuke Koga, Nicholas B. Martin, Dennis W. Dickson

**Affiliations:** ^1^ Department of Neuroscience Mayo Clinic Jacksonville Florida USA; ^2^ Present address: Department of Pathology and Laboratory Medicine Hospital of the University of Pennsylvania Philadelphia Pennsylvania USA

**Keywords:** artificial intelligence, ChatGPT, clinicopathological conference, CPC, Google Bard, large language model, neuropathology, pathology

## Abstract

This study explores the utility of the large language models (LLMs), specifically ChatGPT and Google Bard, in predicting neuropathologic diagnoses from clinical summaries. A total of 25 cases of neurodegenerative disorders presented at Mayo Clinic brain bank Clinico‐Pathological Conferences were analyzed. The LLMs provided multiple pathologic diagnoses and their rationales, which were compared with the final clinical diagnoses made by physicians. ChatGPT‐3.5, ChatGPT‐4, and Google Bard correctly made primary diagnoses in 32%, 52%, and 40% of cases, respectively, while correct diagnoses were included in 76%, 84%, and 76% of cases, respectively. These findings highlight the potential of artificial intelligence tools like ChatGPT in neuropathology, suggesting they may facilitate more comprehensive discussions in clinicopathological conferences.

## Graphical abstract

This study assessed the capability of large language models, namely ChatGPT and Google Bard, in predicting neuropathologic diagnoses from 25 cases presented at Mayo Clinic brain bank clinicopathological conferences. ChatGPT‐4 rendered correct diagnoses in 84% of cases, whereas ChatGPT‐3.5 and Google Bard each achieved 76%. These findings highlight the potential of large language models in neuropathology, suggesting they may facilitate more comprehensive discussions in clinicopathological conferences.

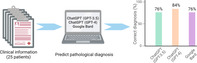

## 1 INTRODUCTION

Artificial Intelligence (AI), specifically large language models (LLMs) like ChatGPT developed by OpenAI, demonstrate promising utility in healthcare applications, from clinical decision support to medical education. As of May 15, 2023, a PubMed search for the term “ChatGPT” yielded 420 manuscripts published in 2023 alone, reflecting the growing interest in AI‐based models in healthcare. A recent study showed that ChatGPT achieved scores at or near the passing threshold across all three stages of the United States Medical Licensing Exam (USMLE) without specialized training [1]. Furthermore, ChatGPT has demonstrated remarkable capabilities in medical education by generating USMLE‐style multiple‐choice questions, hinting at its potential as an effective tool for creating practice question banks [2, 3]. Beyond this, ChatGPT has been found to outperform physicians in online question‐and‐answer exchanges [4]. These findings highlight the potential influence of LLMs in medical education and practice.

Clinicopathological conferences (CPCs) hold significant value in medical education, fostering comprehensive discussions on complex disease processes and their pathologies. Given the complexity and diversity of clinical presentations, these discussions are particularly beneficial for neurodegenerative disorders [5, 6]. Participating in these discussions, however, can often be a daunting task for students and residents due to the complexity of the subject matter.

Previous work employing AI in neuropathology has predominantly focused on image analysis [7, 8, 9]. While these studies contribute significantly to the field, they do not capture the full potential of AI, particularly LLMs, in neuropathology. In the present study, we aimed to examine the ability of LLMs, ChatGPT and Google Bard, to predict neuropathologic diagnoses from clinical summaries presented at CPCs for various neurodegenerative disorders.

## 2 METHODS

This study included 25 cases discussed at the Mayo Clinic brain bank CPC between December 2022 and April 2023. The cases included six cases of Lewy body disease, four each of Alzheimer's disease and multiple system atrophy (MSA), three each of corticobasal degeneration (CBD) and progressive supranuclear palsy (PSP), and one case each of amyotrophic lateral sclerosis, globular glial tauopathy (GGT), Creutzfeldt‐Jakob disease, frontotemporal lobar degeneration with motor neuron disease, and mitochondrial myopathy, encephalopathy, lactic acidosis, and stroke‐like episodes (MELAS). The clinical summary of each patient was prepared by a neuropathologist (DWD). The prompt with each clinical summary was given in ChatGPT‐3.5, ChatGPT‐4, and Google Bard. The prompt was: “Act as a neurologist participating in a Mayo Clinic brain bank clinico‐pathological conference. A summary of the patient's clinical information will be presented, and you will use this information to predict the neuropathological diagnosis (not clinical diagnosis). Describe the multiple differential diagnoses and the rationale for each. Please list the disease you consider most likely at the top.”

An example of the clinical summary (Case 6) is provided as follows: “This 61‐year‐old man had about a 4‐year history of parkinsonism, as well as memory problems and forgetfulness. He had a shuffling gait, poor posture, and poor balance with frequent falls backwards. He had difficulty manipulating objects with both hands. He also had intermittent resting and action tremors of his hands. He had erectile dysfunction, urinary frequency, and urinary incontinence. He had occasional syncope. He had personality changes and became apathetic and withdrawn. He developed dream enactment behaviors, including screaming and thrashing and falling out of bed. Slowness and balance worsened, and he developed freezing of gait. His past medical history was notable for obstructive sleep apnea. His family history was notable for dementia in both parents and a maternal aunt, as well as Parkinson's disease in his paternal grandmother. The neurologic exam was notable for ideomotor apraxia of the left hand. His speech had low volume, reduced rate, and reduced prosody. He had severe bradyphrenia. He had a grasp reflex. His cranial nerve exam was notable for severe limitation of vertical eye movements that was consistent with supranuclear palsy. Other cranial nerves were normal except for hypomimia. He had mild axial rigidity in arms more than legs, worse on the left. There was no dystonia. He had stimulus‐sensitive myoclonus in his fingers with arms outstretched. He had no tremor at rest, but a mild postural and mild kinetic tremor. His coordination was mildly impaired in hands and feet. He had no dysmetria on finger‐to‐nose testing. He needed two‐person assistance to rise from a chair. He had severe anterocollis. He had a wide‐based gait, reduced speed, reduced stride length and reduced arm swing. An MRI showed moderate to severe cortical atrophy, predominantly affecting the frontal and temporal lobes. A genetic screen with a panel for Parkinson's disease, amyotrophic lateral sclerosis, and frontotemporal dementia genes was negative.”

## 3 RESULTS AND DISCUSSION

ChatGPT‐3.5, ChatGPT‐4, and Google Bard, provided multiple pathologic diagnoses (Table 1) and their rationale. Table 1 also includes the final clinical diagnoses made by the physicians for comparison. Overall, 32% of primary diagnoses made by ChatGPT‐3.5, 52% by ChatGPT‐4, and 40% by Google Bard aligned with the neuropathological diagnoses made by the neuropathologist. Correct diagnoses were included in 76% of cases for ChatGPT‐3.5, 84% for ChatGPT‐4, and 76% for Google Bard.

**TABLE 1:** The pathological diagnoses by pathologist and ChatGPT.

Case #	Pathological Dx	Clinical Dx	GPT‐4: Primary and other diagnoses	GPT‐3.5: Primary and other diagnoses	Google Bard: Primary and other diagnoses
1	AD	CBD	CBD, AD, VaD, FTD	CBD, AD, FTD, DLB	AD, FTD, PD, VaD, Lewy body dementia
2	AD	pseudo‐dementia	AD, VaD, Lewy body dementia, FTD, Prion disease	DLB, AD, VaD, CJD	AD, VaD, Lewy body dementia, CJD, Severe depression with psychotic features
3	AD	AD & VaD vs DLB	AD, Lewy body dementia, VaD, FTD	AD with cerebrovascular disease, AD, VaD, DLB, Mixed AD and vascular pathology	AD, VaD, Lewy body dementia, Mixed dementia, FTD
4	AD	LBD vs VaD	Lewy body dementia, AD, VCI/VaD, Coexistence of LBD and VCI/VaD or AD	PDD, LBD, VaD, AD	Lewy body dementia, AD, VaD, PDD
5	ALS	ALS	ALS, FTD‐MND, PLS, Multifocal Motor Neuropathy	ALS with FTD, ALS, PLS, HSP, MND associated with malignancy	ALS, PLS, FTD, MSA
6	CBD	PSP vs DLB	PSP, MSA, CBD, DLB	PSP, MSA, CBD, AD	PDD, PSP, CBD, MSA
7	CBD + AD	PPA	AD, PPA, logopenic, CBD, PSP	AD, PPA, CBS	FTD, VaD, PDD
8	CBD + AD	AD & Logopenic aphasia	AD, VaD, Lewy body dementia, FTD	AD, VaD	AD, VaD, FTD
9	CJD	CJD	CJD, vCJD, AE, Prion disease, unspecified	CJD, vCJD, AE, FTD, AD	CJD, FTD, PD
10	LBD	DLB	DLB, PDD, AD, VaD	DLB, AD, PDD	AD, PD, Lewy body dementia
11	LBD	DLB	Lewy body dementia, PDD, MSA, AD, VCI/VaD	DLB with cerebral microbleeding and acute intracerebral hematoma, DLB, VaD, AD, Cerebrovascular disease	VaD, AD, PDD
12	LBD	DLB	DLB, AD, VaD, PDD	AD, mild cognitive impairment, PDD, NPH	AD, VaD, FTD
13	LBD	PD	PSP, MSA, CBD	MSA, PD, DLB, PSP	PD, VaP, PSP
14	LBD	LBD	DLB, AD, PDD, FTD	DLB, AD, PDD, FTD	AD, Lewy body dementia, FTD
15	LBD	LBD	DLB, AD, VaD, CBD	AD, VaD, FTD	AD, VaD, Lewy body dementia
16	FTLD‐MND	FTD‐MND	FTLD, AD, VaD, DLB	bvFTD, AD, Lewy body dementia, PPA, VaD	FTD, AD, VaD
17	GGT	PiD & PPA	PPA, VaD, AD, FTD	PPAOS, AD, PDD, DLB	PPA, PNFA, SD
18	MELAS	MS vs autoimmune encephalitis	Prion disease, MS, AE, Leukoencephalopathy or other white matter disease, Mitochondrial or other metabolic disorders, Primary or Secondary CNS Vasculitis	Prion disease, CJD, vCJD, other prion diseases	CJD, PML, MS
19	MSA	MSA	PD, MSA, ET	PD, MSA, PSP, AD	MSA, PD, PSP
20	MSA	MSA	PD, PSP, MSA	PD, MSA, PSP	PD, PSP, MSA
21	MSA	PSP vs CBD vs MSA	CBD, PSP, PD, MSA	PSP	PSP, MSA, CBD
22	MSA‐C	MSA	MSA‐C, SCA, PD plus syndrome	MSA, PD, DLB, CBD, PSP	MSA, SCA, FA
23	PSP	CBD	CBD, PLS, PD plus syndrome	PSP, PD, MSA, CBD	PPA, PSP, CBD
24	PSP	CBD	CBD, PDD, PSP	PSP, PD, MSA, CBD	PD, PSP, MSA
25	PSP	PSP	PSP, CBD, MSA, PD	MSA, PDD, PSP, CBD	PSP, CBD, PD

*Note*: Of three CBD cases, two have concurrent AD; however, we consider only CBD correct answer. For cases of LBD, we consider either DLB, PDD, or Lewy body dementia correct answers.

Abbreviations: AD, Alzheimer's disease; AE, Autoimmune encephalitis; ALS, amyotrophic lateral sclerosis; bvFTD, behavioral variant frontotemporal dementia; CBD, corticobasal degeneration; CJD, Creutzfeldt‐Jakob disease; vCJD, variant, Creutzfeldt‐Jakob disease; DLB, dementia with Lewy bodies; Dx, diagnosis; ET, essential tremor; FA, Friedreich's ataxia; FTD, frontotemporal dementia; FTLD, frontotemporal lobar degeneration; GGT, globular glial tauopathy; HSP, Hereditary spastic paraplegia; LBD, Lewy body disease; MELAS, mitochondrial myopathy, encephalopathy, lactic acidosis, and stroke‐like episodes; MND, motor neuron disease; MS, multiple sclerosis; MSA, multiple system atrophy; MSA‐C, multiple system atrophy cerebellar type; NPH, normal pressure hydrocephalus; PD, Parkinson's disease; PDD, PD with dementia; PLS, primary lateral sclerosis; PPA, primary progressive aphasia; PPAOS primary progressive aphasia of speech; PSP, progressive supranuclear palsy; SCA, spinocerebellar ataxia; VaD, Vascular dementia.

The clinical summary provided for Case 6 offers an illustrative case study. The neuropathological diagnosis of Case 6 was CBD, but ChatGPT‐3.5 and ChatGPT‐4 answered PSP as the most likely diagnosis, followed by MSA and CBD. Google Bard, on the other hand, answered PD with dementia as the most probable diagnosis, followed by PSP, CBD, and MSA. These differential diagnoses seem reasonable based on the clinical information. Parkinsonism, poor balance with frequent falls, and supranuclear palsy were suggestive of PSP, while autonomic dysfunction and anterocollis were suggestive of MSA. Ideomotor apraxia of the left hand and myoclonus were considered corticobasal syndrome, a typical clinical presentation of CBD. Interestingly, the final clinical diagnosis made by the physician was PSP versus dementia with Lewy bodies, which highlights the reasonable nature of the differential diagnoses suggested by these LLMs.

The results demonstrate that LLMs can predict pathological diagnoses from clinical information with reasonable accuracy. Even though the correct pathologic diagnosis was not included in some cases, a similar scenario may occur with human physicians, as predictions depend heavily on the quality and specificity of clinical summaries. Moreover, predicting the pathologic diagnosis from clinical information in some disorders, such as GGT, is highly challenging, even for experts [10]. This difficulty was exemplified in Case 17, where both the LLMs and the clinicians (who diagnosed Pick's disease and primary progressive aphasia) failed to correctly identify the pathology as GGT. Another challenge encountered during the study was that the models occasionally proposed diagnoses that were more clinical (e.g., frontotemporal dementia) than neuropathological (e.g., frontotemporal lobar degeneration), despite explicit instructions to avoid clinical diagnoses.

One limitation of our study is that while we compared the LLMs' predictions to the final clinical diagnoses made by the physicians who had direct clinical interaction with the patients, we did not compare the LLMs' predictions directly with predictions made by physicians given the same clinical summaries. This comparison in a prospective study would allow us to assess the diagnostic performance of the LLMs versus that of human physicians under the same conditions. Furthermore, due to the small sample size, we did not perform a formal statistical comparison between the diagnostic accuracy of the three models, although the results suggest a potential superiority of ChatGPT‐4 over ChatGPT‐3.5 and Google Bard. This observation warrants further investigation in future research with larger sample sizes and rigorous statistical analysis. The focus of this study solely on neurodegenerative disorders is another limitation, which may influence the generalizability of the results.

In conclusion, LLMs can generate an adequate range of possible neuropathologic diagnoses based on the clinical summary. This finding suggests that LLMs can facilitate discussions by novice participants in CPCs who may not be familiar with neuropathological diagnoses. The insights derived from this study contribute to the ongoing controversy on the role and effectiveness of AI models in medical education and clinical practice.

## AUTHOR CONTRIBUTIONS

Shunsuke Koga developed the original concept, provided supervision for the project, performed formal analysis, and drafted the manuscript. Nicholas B. Martin assisted in the preparation of the data. Dennis W. Dickson conducted the pathological diagnosis and edited the manuscript. All authors reviewed and approved the final manuscript.

## CONFLICT OF INTEREST STATEMENT

The authors declare no conflicts of interest.

## Data Availability

The data that support the findings of this study are available from the corresponding author upon reasonable request.
